# Editorial: The contrast sensitivity function: from laboratory to clinic, volume II

**DOI:** 10.3389/fnins.2023.1336649

**Published:** 2023-12-05

**Authors:** Fang Hou, Zhong-Lin Lu, Peter Bex, Alexandre Reynaud

**Affiliations:** ^1^State Key Laboratory of Ophthalmology, Optometry and Vision Science, Eye Hospital, Wenzhou Medical University, Wenzhou, China; ^2^National Engineering Research Center of Ophthalmology and Optometry, Eye Hospital, Wenzhou Medical University, Wenzhou, China; ^3^School of Ophthalmology and Optometry, Wenzhou Medical University, Wenzhou, China; ^4^Division of Arts and Sciences, NYU Shanghai, Shanghai, China; ^5^Center for Neural Science and Department of Psychology, New York University, New York, NY, United States; ^6^NYU-ECNU Institute of Brain and Cognitive Science at NYU Shanghai, Shanghai, China; ^7^Department of Psychology, Northeastern University, Boston, MA, United States; ^8^McGill Vision Research, McGill University, Montréal, QC, Canada

**Keywords:** contrast sensitivity function, spatial frequency channel, binocular summation, monocular deprivation, myopia, ortho-K, thyroid-associated ophthalmopathy

This is the second volume of Research Topic, “*The contrast sensitivity function: from laboratory to clinic*”.

Visual acuity evaluates spatial resolution (Whittaker and Lovie-Kitchin, 1993), but it is a poor predictor of performance in critical daily tasks (Gruber et al., 2013). On the other hand, contrast sensitivity (1/threshold) measures the ability to detect subtle changes in light against a background. The contrast sensitivity function (CSF) quantifies how contrast sensitivity varies as a function of spatial frequency, accounting for optical and neural effects at various stages in the central visual system. Importantly, the CSF is more closely related to performance in daily visual tasks (Owsley et al., 2020).

Many visual diseases affect visual acuity and the CSF differently. In the early stages of several clinical conditions, visual acuity may remain normal or near-normal, while the CSF exhibits deficits that may be spatial frequency dependent (Vingopoulos et al., 2023). Moreover, several studies found significant structure-function associations between CSF deficits and anatomical changes in various eye diseases (Tu et al., 2023).

This Research Topic comprises six research articles. The first three articles focus on understanding the visual system. Visual information is initially processed by a bank of spatial frequency channels beginning in the retina and continuing through primary visual cortex. Reynaud and Min applied exploratory factor analysis to contrast sensitivity data from amblyopic and normally-sighted individuals in five different studies. They discovered that the CSF in the amblyopic visual system is subserved by the same spatial frequency channels as the normal visual system, with the only difference being an approximately 50% reduction in the weight attributed to the high-spatial frequency channel in the amblyopic eye.

Anatomically, the eyes provide two separate streams of information, which are integrated into a binocular response by the primary visual cortex. Yu and Watson investigated binocular summation at high and low contrasts in visual acuity measurement and found a positive correlation in binocular summation between high and low contrast. They also identified a significant association between a baseline measure and the change in binocular summation between contrast levels.

Monocular pattern deprivation (MPD) can modulate binocular interactions (Hess and Min, 2023). Li et al. found MPD effectively improved the contrast sensitivity of the deprived eye, especially when external noise was absent, and had a more profound effect at higher spatial frequencies. Using the perceptual template model (PTM) (Lu and Dosher, 1998), their results revealed that MPD decreased internal additive noise in the deprived eye, providing insights into the mechanisms underlying short-term MPD.

The remaining studies demonstrated the clinical utility of measuring the CSF in different eye diseases. In the study by Xu et al., the central and peripheral CSF were measured in myopic and emmetropic individuals. Myopic individuals exhibited significantly increased contrast sensitivity in the 6° parafovea and 12° parafovea regions compared to emmetropic individuals. The authors suggested that peripheral contrast sensitivity may play a role in the growth of emmetropic eyes.

Lu et al. evaluated the changes in optical quality and visual function after 3 months of wearing orthokeratotic (OK) lenses in children. They observed that optical quality decreased after 3 months of OK lens wear, while the CSF remained unchanged. This suggests that neural adaptation could compensate for the alteration in optical quality.

Jin et al. measured S-cone sensitivity in patients with thyroid-associated ophthalmopathy (TAO) who had normal visual acuity and visual fields, and no apparent signs of orbital congestion. They found a selective S-cone deficit in the early stage of TAO, indicating that S-cone sensitivity can be used for early detection of thyroid dysfunction optic neuropathy in TAO patients.

In summary, in addition to our first volume of “*The contrast sensitivity function: from laboratory to clinic*” (Hou et al., 2021), current Research Topic not only enriches our understanding of visual processing but also provides valuable insights into the clinical management of eye diseases.

## Author contributions

FH: Writing—original draft, Writing—review & editing. Z-LL: Writing—review & editing. PB: Writing—review & editing. AR: Writing—review & editing.
